# 587 Pre-admission MRSA Status Has a Profound Impact on Our Burn Patients

**DOI:** 10.1093/jbcr/iraf019.216

**Published:** 2025-04-01

**Authors:** Michael Feldman, Tiffany Lord, Mark Altman, Prabhu Senthil-Kumar

**Affiliations:** Virginia Commonwealth University; Virginia Commonwealth University; Evans-Haynes Burn Center; Evans-Haynes Burn Center

## Abstract

**Introduction:**

Burn patients are well known to be at risk for infection. The presence of multi drug resistant bacteria, methicillin-resistant staphylococcus aureus in particular, has been linked to increased complications in the burn community. We have created a quality improvement project looking at the presence and impact of MRSA on our burn patients.

**Methods:**

This represents a quality improvement project. Data was collected from our burn registry. Data included age, length of stay, intensive care unit length of stay, ventilator days, mechanism of injury, and complications. We also characterize specific measures to help improve ordering and collecting MRSA screens during this period of time.

**Results:**

We reviewed 321 patients over a 6-month period. Of those, 5 pediatric and 26 adult patients were noted to have MRSA on admission. MRSA positive patients had increased length of stay (12.8 versus 7.7) and increased time in the ICU (11 versus 8.8 days). This was despite the Non-MRSA community having a higher overall average ventilator days (11.1 versus 9.6 days). In addition, our MRSA positive patients were noted to have an increase in total number of complications versus their Non-MRSA counterparts.

This information is particularly important as we have documented a sharp increase in MRSA positive patients from the community (recently 18% versus a range of 2.6% to 6.7%). Compliance with both ordering and collecting MRSA labs was greater than 95% on average. We accomplished this by automating orders within our admission orderset, reviewing all admissions for compliance and providing real time feedback through Performance Improvement meetings and a burn manual.

**Conclusions:**

Multidrug resistant bacteria, MRSA in particular, have a profound impact on our burn patients. In particular it has been linked to an increased LOS, time in the ICU and complication rates. We would advocate for collecting this information and using it to inform and treat burn patients. This is particularly important as the number of MRSA positive patients is increasing in our community. The process of collecting these samples is improved through the use of automation within the EMR and feedback to the burn team.

**Applicability of Research to Practice:**

MRSA status is an important part of burn patient management. This information can help predict outcomes. Collecting this information requires a systematic approach.

**Funding for the Study:**

N/A

